# Clinical performance of subcutaneous vs. transvenous implantable defibrillator in patients with ischemic cardiomyopathy: *data from Monaldi Rhythm Registry*

**DOI:** 10.3389/fcvm.2025.1539125

**Published:** 2025-02-19

**Authors:** Vincenzo Russo, Alfredo Caturano, Valter Bianchi, Anna Rago, Ernesto Ammendola, Andrea Antonio Papa, Nadia Della Cioppa, Annamaria Guarino, Alessandro Masi, Antonio D'Onofrio, Paolo Golino, Emilio Di Lorenzo, Gerardo Nigro

**Affiliations:** ^1^ Division of Cardiology, Department of Medical Translational Sciences, University of Campania Luigi Vanvitelli, Naples, Italy; ^2^ Department of Advanced Medical and Surgical Sciences, University of Campania Luigi Vanvitelli, Naples, Italy; ^3^ Department of Human Sciences and Promotion of the Quality of Life, San Raffaele Roma Open University, Rome, Italy; ^4^ Departmental Unit of Electrophysiology, Evaluation and Treatment of Arrhythmias, Monaldi Hospital, Naples, Italy; ^5^ Department of Anesthesiology, Monaldi Hospital, Napoli, Italy; ^6^ Department of Cardiology, AORN dei Colli-Monaldi Hospital, Naples, Italy

**Keywords:** subcutaneous ICD (S-ICD), transvenous ICD, complications, infections, inappropriate shock therapy, ischemic cardiomyopathy

## Abstract

**Introduction:**

Subcutaneous ICD (S-ICD) is an alternative to a transvenous implantable cardioverter-defibrillator (TV-ICD) system in selected patients not in need of pacing or resynchronization. Currently, little is known about the effectiveness and safety of S-ICD in patients with ischemic cardiomyopathy (ICM). The aim of our study was to describe the clinical features and the drivers of S-ICD implantation among patients with ICM, as well as the clinical performance of S-ICD vs. TV-ICD among this subset of patients during a long-term follow-up.

**Materials and methods:**

All ICM patients with both S-ICD and TV-ICD implanted and followed at Monaldi Hospital from January 1, 2015, to January 1, 2024, were evaluated; among them, only ICD recipients with no pacing indication were included. We collected clinical and anamnestic characteristics, as well as ICD inappropriate therapies, ICD-related complications and infections.

**Results:**

A total of 243 ICM patients (mean age 63.0 ± 11.0, male 86.0%) implanted with TV-ICD (*n:* 129, 53.1%) and S-ICD (*n:* 114, 46.9%) followed at our center for a median follow-up of 66.9 [39.4–96.4] months were included in the study. Kaplan–Meier analysis revealed no significant difference in the risk of inappropriate ICD therapies (log-rank *p* = 0.137) or ICD-related complications (log-rank *p* = 0.055) between S-ICD and TV-ICD groups. TV-ICD patients showed a significantly higher risk of ICD-related infections compared to those in the S-ICD group (log-rank *p* = 0.048). At multivariate logistic regression analysis, the only independent predictors of S-ICD implantation were female sex [OR: 52.62; *p* < 0.001] and primary prevention [*OR:* 17.60; *p* < *0.001*].

**Conclusions:**

Among patients with ICM not in need of pacing or resynchronization (CRT), the decision to implant an S-ICD was primarily influenced by female gender and primary prevention indications. No significant differences in inappropriate ICD therapies and complications were found; in contrast, the S-ICD group showed a numerically reduced risk of ICD-related infections.

## Introduction

Ischemic cardiomyopathy (ICM) is currently defined a myocardial disease characterized by impaired systolic left ventricle ejection fraction (LVEF) in the setting of obstructive coronary artery disease (CAD) and represents the most common cause of heart failure (1). Implantable cardioverter defibrillator (ICD) is effective for the primary prevention of sudden cardiac death (SCD) among ICM patients (2). Subcutaneous ICD (S-ICD) is an alternative to a transvenous implantable cardioverter-defibrillator (TV-ICD) system in selected patients not in need of pacing or CRT (3–10).

Currently, no sub-analysis of randomized clinical trials including ICM patients are available and real-world data comparing the effectiveness and safety of S-ICD in this clinical setting are lacking (11). The aim of our study was to describe the clinical features and the drivers of S-ICD implantation among patients with ICM, as well as the clinical performance of S-ICD vs. TV-ICD among this subset of patients during a long-term follow-up.

## Materials and methods

This is a single-center, retrospective observational study. Data for this study were sourced from Monaldi Hospital Rhythm Registry (NCT05072119), which includes all patients who underwent ICD implantation and followed up at our Institution through both outpatient visits, every 3–6 months, and remote device monitoring. During the follow-up, the occurrence and the causes of inappropriate and appropriate ICD therapies, and ICD-related complications were assessed and recorded in the electronic data management system. For the present analysis, we selected all consecutive patients with ICM who received subcutaneous (S-ICD Group) and transvenous (TV-ICD Group) in primary or secondary prevention, from January 1, 2015 to January 1, 2024, according to the European guidelines and recommendations available at the time of implantation (12, 13). Only patients not in need of pacing or CRT that underwent TV-ICD implantation were included in the analysis. At our center, the choice between S-ICD and TV-ICD is guided by a shared decision-making process, which includes both implanting physician and patient preference. All S-ICDs were implanted under deep sedation and using the intermuscular two-incision technique (14, 15). The local institutional review boards approved the study (ID 553-19), and all patients provided written informed consent for data storage and analysis.

### ICD programming

The programming of the parameters for the detection of ventricular tachycardia (VT) or ventricular fibrillation (VF) was done according to the guideline's recommendations at the time of the implant. We routinely activate for primary prevention only one VF zone (30 intervals at 250 bpm) and for secondary prevention two windows of detection (VF: 30 intervals at 250 bpm; VT2: 30 intervals at 187 bpm or 10–20 bpm < VT rate) with shocks and up to three anti-tachycardia pacing (ATP) and eight shocks in VT2 zone. S-ICD devices were programmed with a conditional zone, between 200 and 250 bpm, and a shock zone > 250 bpm. The programmed sensing vector was primary (60.3%) or secondary (37.5%) for most patients and alternate in small percentage of cases (2.2%).

### Outcomes

**Figure 1:**
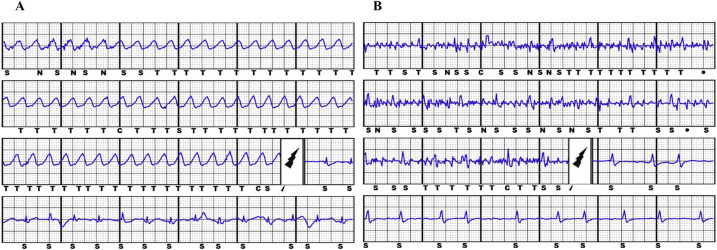
Example of appropriate S-ICD shock due to ventricular fibrillation (panel **A**) and inappropriate S-ICD shock due to muscular noise (panel **B**).

The primary study endpoints were ICD inappropriate therapies, defined as ATP and/or shocks for conditions other than VT/VF (Figure 1); ICD-related complications, defined as peri-procedural implantation complications, pulse generator or lead-related complications, infections which required complete removal of the system. The secondary endpoints were the clinical variables associated to S-ICD implantation.

### Statistical analysis

Categorical data were expressed as number and percentage, whereas continuous variables were expressed as either median [interquartile range (IQR)] or mean ± standard deviation (SD), based on their distribution. Between-group differences for categorical variables were assessed using the chi-square test, with Yates' correction applied where appropriate. Continuous variables were compared using either the parametric Student's *t*-test or the nonparametric Mann–Whitney *U* test, depending on their distribution. Kaplan–Meier analysis was performed to evaluate the main outcomes of interest, stratified by ICD type, with survival curves compared using the log-rank test. Cox proportional hazards univariate and multivariate regression was used to assess the relationship between the variables of interest and the risk of adverse outcomes. Additionally, univariate and multivariate logistic regression analysis was conducted to identify baseline characteristics associated with S-ICD implantation. All analyses were performed using RStudio software (RStudio, Boston, MA).

## Results

### Study population

A total of 243 ICM patients (mean age 63.0 ± 11.0, male 86.0%) with TV-ICD (*n:* 129, 53.1%) and S-ICD (*n:* 114, 46.9%) followed at our center for a median follow-up of 66.9 [39.4–96.4] months were included in the study. The indication for ICD implantation was primary prevention in 189 patients (77.8%) and secondary prevention in 54 patients (22.2%). S-ICD patients were younger (61.0 ± 11.0 vs. 65.9 ± 10.5 years, *p* *<* *0.001*) and showed less frequently hypertension (61.4% vs. 81.4%, *p* *=* *<0.001*) and diabetes (19.3% vs. 41.9%, *p* *=* *<0.001*). The baseline clinical characteristics of the study population are summarized in Table 1. At multivariate logistic regression analysis, the only independent predictors of S-ICD implantation were female sex [odds ratio (OR): 52.62; 95% confidence interval (CI) 20.23–136.83; *p* *<* *0.001*] and primary prevention [OR*:* 17.60; 95% CI 5.30–58.38; *p* < *0.001*] (Table 2). Regarding S-ICD group, no patients required device extraction due to the need for pacing or CRT.

**Table 1:** Baseline characteristics of the study population divided according to the ICD type.

Variable	Overall (*n* = 243)	S-ICD group (*n* = 114)	TV-ICD group (*n* = 129)	*p*
Age, y, mean (SD)	63.0 ± 11.0	61.0 ± 11.0	65.9 ± 10.5	<0.001
Male gender, *n* (%)	209 (86.0%)	101 (88.6)	108 (83.7)	0.274
NYHA I, *n* (%)	7 (2.9)	7 (6.1)	–	0.019
NYHA II, *n* (%)	111 (45.7)	47 (41.2)	64 (49.6)	0.191
NYHA III, *n* (%)	94 (38.7)	41 (36.0)	53 (41.1)	0.412
NYHA IV, *n* (%)	14 (5.8)	2 (1.8)	12 (9.3)	0.011
LVEF (%), median [IQR]	30 [25.0–35.0]	30 ± 8	30.0 [25.0–35.0]	0.085
History of CAD, *n* (%)	208 (85.6)	100 (87.7)	108 (83.7)	0.370
Recent MI, *n* (%)	191 (78.6)	98 (86.0)	93 (72.1)	0.008
Previous CABG, *n* (%)	43 (17.7)	23 (20.2)	20 (15.5)	0.340
Previous PTCA, *n* (%)	139 (57.2)	60 (52.6)	79 (61.2)	0.173
PAD, *n* (%)	29 (11.9)	11 (9.6)	18 (14.0)	0.303
Previous stroke/TIA, *n* (%)	11 (4.5)	4 (3.5)	7 (5.4)	0.470
Previous valve replacement, *n* (%)	15 (6.2)	6 (5.3)	9 (7.0)	0.570
AF history, *n* (%)	65 (26.8)	38 (33.3)	27 (20.9)	0.020
Hypertension, *n* (%)	175 (72)	70 (61.4)	105 (81.4)	<0.001
Diabetes, *n* (%)	76 (31.3)	22 (19.3)	54 (41.9)	<0.001
COPD, *n* (%)	62 (25.5)	32 (28.1)	30 (23.3)	0.780
CKD, *n* (%)	57 (23.5)	26 (22.8)	31 (24.0)	0.820
Primary prevention, *n* (%)	189 (77.8)	107 (93.9)	82 (63.6)	<0.001
Secondary prevention, *n* (%)	54 (22.2)	7 (6.1)	47 (36.4)	<0.001
Follow-up months, median [IQR]	66.9 [39.4–96.4]	70.7 [36.1–101.4]	60.5 [24.2–91.0]	0.694

ICD, implantable cardioverter defibrillator; S-ICD, subcutaneous implantable cardioverter defibrillator; TV-ICD, transvenous implantable cardioverter defibrillator; SD, standard deviation; LVEF, left ventricular ejection fraction; MI, myocardial infarction; CAD, coronary artery disease; CABG, coronary artery bypass graft; PTCA, percutaneous transluminal coronary angioplasty; PAD, peripheral artery disease; TIA, transient ischemic attack; AF, atrial fibrillation; COPD, chronic obstructive pulmonary disease; CKD, chronic kidney disease; ATP, anti-tachycardia pacing; PG, pulse generator.

**Table 2:** Univariable and multivariable logistic regression model for S-ICD implantation choice.

Variable	Univariable analysis				Multivariable analysis			
	OR	95% CI		*p*	OR	95% CI		*p*
Age	0.97	0.95	0.99	0.027	1.01	0.97	1.06	0.595
Sex								
M	1				1			
F	39.96	19.01	84.00	<0.001	52.62	20.23	136.83	<0.001
Primary prevention	8.76	3.77	20.39	<0.001	17.60	5.30	58.38	<0.001
Secondary prevention	0.11	0.05	0.27	<0.001				
NYHA class >2	0.62	0.41	0.93	0.022	0.50	0.23	1.05	0.066
LVEF	0.98	0.95	1.01	0.288				
Hypertension	0.36	0.20	0.65	<0.001	0.90	0.33	2.47	0.837
Diabetes	0.33	0.19	0.59	<0.001	0.52	0.49	1.44	0.210
COPD	1.29	0.72	2.29	0.391				
CAD history	1.04	0.52	2.06	0.917				
Previous CABG	1.38	0.71	2.67	0.342				
Previous PTCA	0.72	0.43	1.20	0.202				
History of stroke/TIA	0.63	0.18	2.22	0.476				
PAD	0.66	0.30	1.46	0.304				
CKD	0.93	0.52	1.69	0.822				
AF history	1.89	1.06	3.36	0.030	1.62	0.51	5.15	0.538
Previous valve replacement	0.74	0.26	2.15	0.581				

ICD, implantable cardioverter defibrillator; S-ICD, subcutaneous implantable cardioverter defibrillator; LVEF, left ventricular ejection fraction; COPD, chronic obstructive pulmonary disease; CAD, coronary artery disease; CABG, coronary artery bypass graft; PTCA, percutaneous transluminal coronary angioplasty; TIA, transient ischemic attack; PAD, peripheral artery disease; CKD, chronic kidney disease; AF, atrial fibrillation.

### Clinical outcomes

Among our study population, ICD inappropriate therapies were experienced by 6 patients (2.5%); of them, 2 (1.6%) in S-ICD group and 4 (3.5%) in the TV-ICD group (*p* = 0.327) (Table 3).

**Table 3:** Clinical outcome events among study population.

	Overall (*n* = 243)	S-ICD group (*n* = 114)	TV-ICD group (*n* = 129)	*p*
Appropriate therapies, *n* (%)	21 (8.6)	3 (2.6)	18 (13.9)	0.002
ICD shock, *n* (%)	13 (5.3)	3 (4.3)	10 (7.7)	
ATP, *n* (%)	8 (3.3)	–	8 (6.2)	
Inappropriate therapies, *n* (%)	6 (2.5)	2 (1.8)	4 (3.1)	
Atrial fibrillation, *n* (%)	4 (1.6)	1 (0.9)	3 (2.3)	
T wave oversensing, *n* (%)	1 (0.4)	–	1 (0.8)	
Air entrapment, *n* (%)	1 (0.4)	1 (0.9)	–	0.327
ICD-related complications, *n* (%)	9 (3.7)	2 (1.8)	7 (5.4)	0.131
PG malfunction, *n* (%)	2 (0.9)	2 (1.8)	–	
Lead complications, *n* (%)	7 (3.1)	–	7 (5.4)	
ICD-related infections, *n* (%)	8 (3.3)	1 (0.9)	7 (5.4)	0.048

ICD, implantable cardioverter defibrillator; ATP, anti-tachycardia pacing; PG, pulse generator.

**Figure 2:**
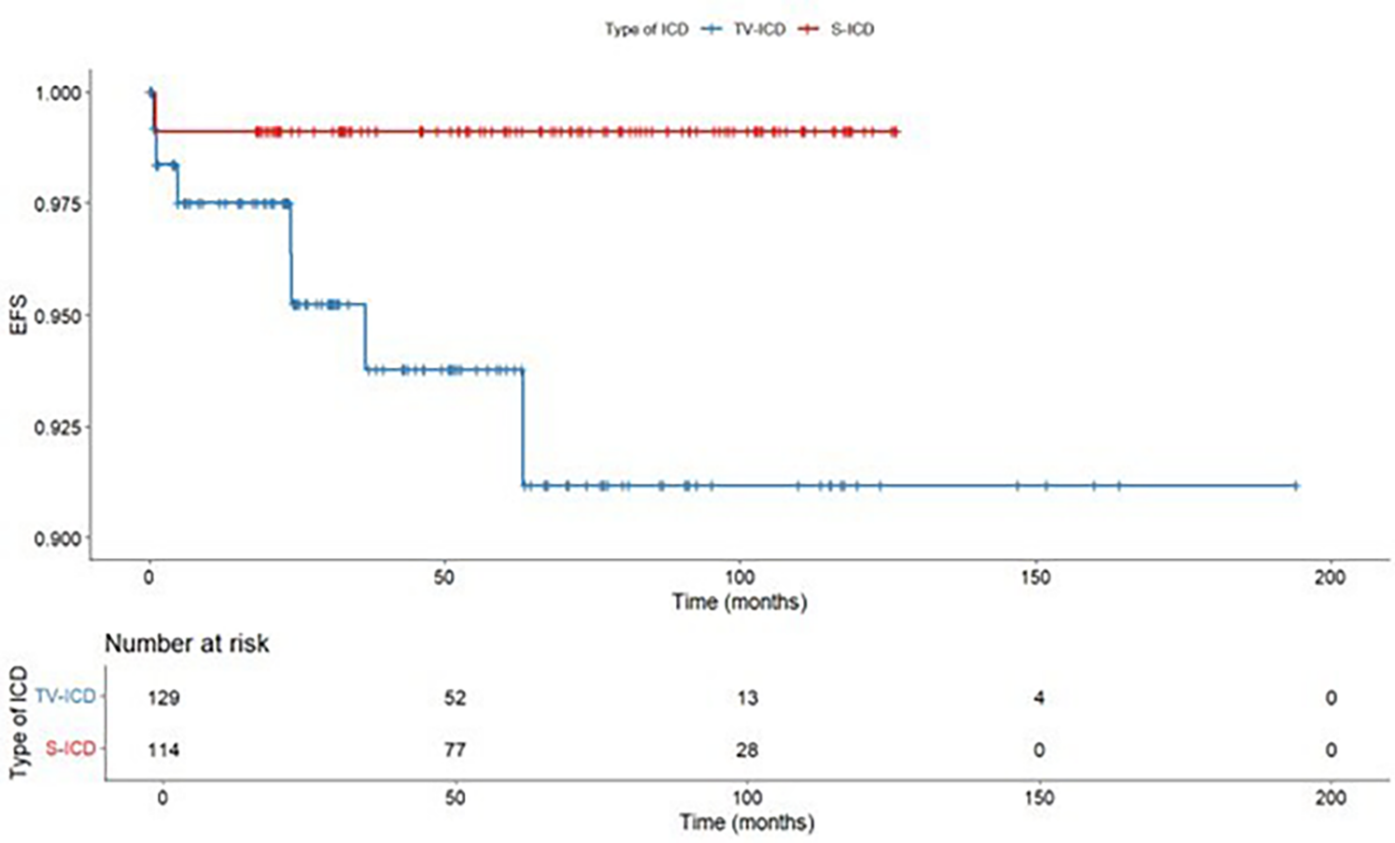
Kaplan maier curve for inappropriate ICD therapies stratified according to ICD type.

The annual incident rate of ICD inappropriate therapies over 5 years was 0.4%. The Kaplan–Meyer analysis did not show a significantly different risk of inappropriate ICD therapies between the two subgroups (log-rank *p* = 0.319) (Figure 2). At Cox multivariate analysis no patients' clinical characteristic, including S-ICD, was associated with inappropriate ICD therapies (Supplementary Table S1).

**Figure 3:**
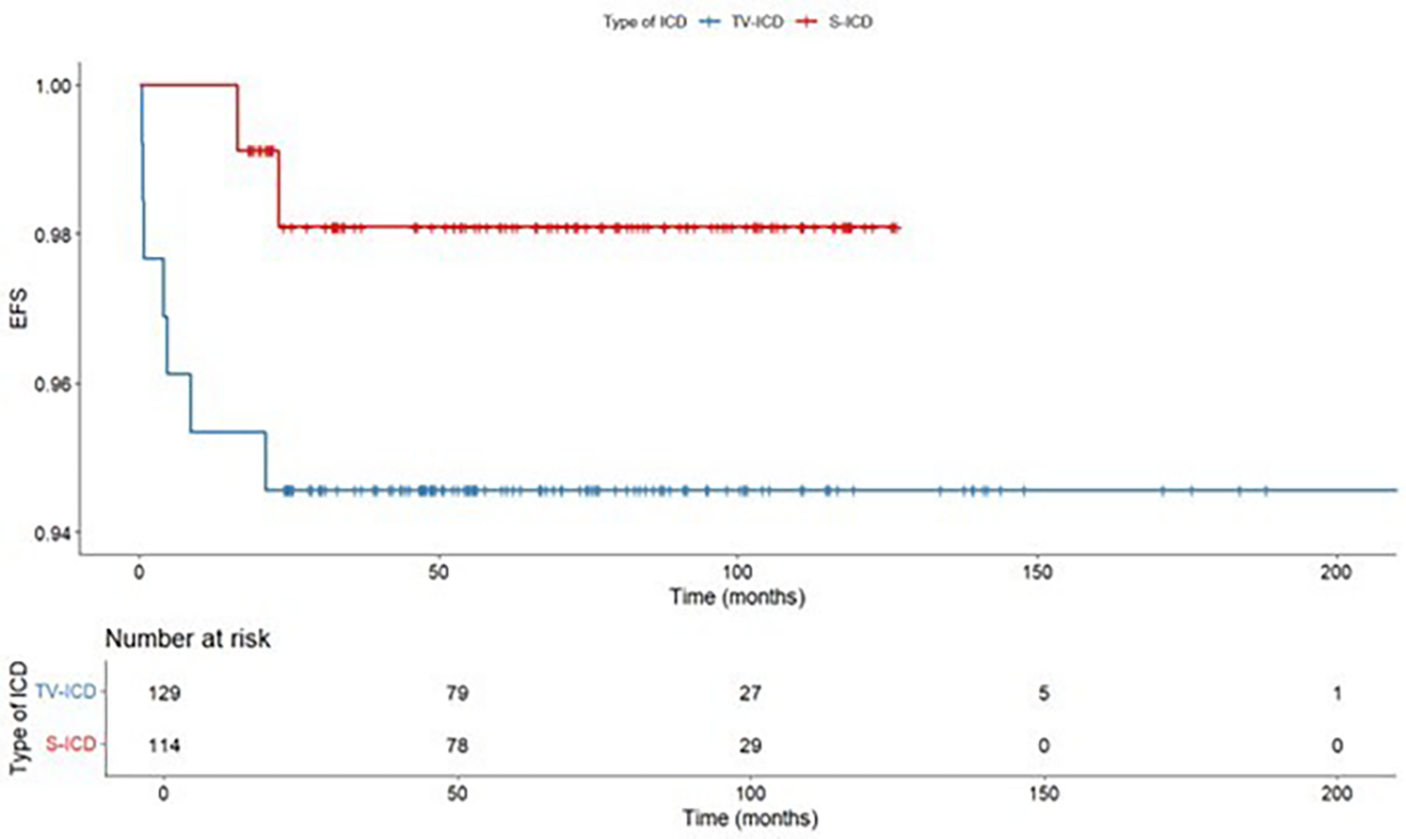
Kaplan maier curve for ICD related complications stratified according to ICD type.

ICD related complications requiring surgical revision occurred in 9 patients (3.7%), of them, 2 (1.8%) in S-ICD group and 7 (5.4%) in the TV-ICD group (*p* = 0.131) (Table 3). S-ICD complications were exclusively attributed to PG malfunctions, whereas all TV-ICD complications were associated with lead-related issues. The Kaplan–Meier analysis did not show a significantly different risk of ICD related complications between the two subgroups (log-rank *p* = 0.137) (Figure 3). At Cox multivariate analysis no patients' clinical characteristics, including S-ICD, was associated with ICD complications (Supplementary Table S2).

**Figure 4:**
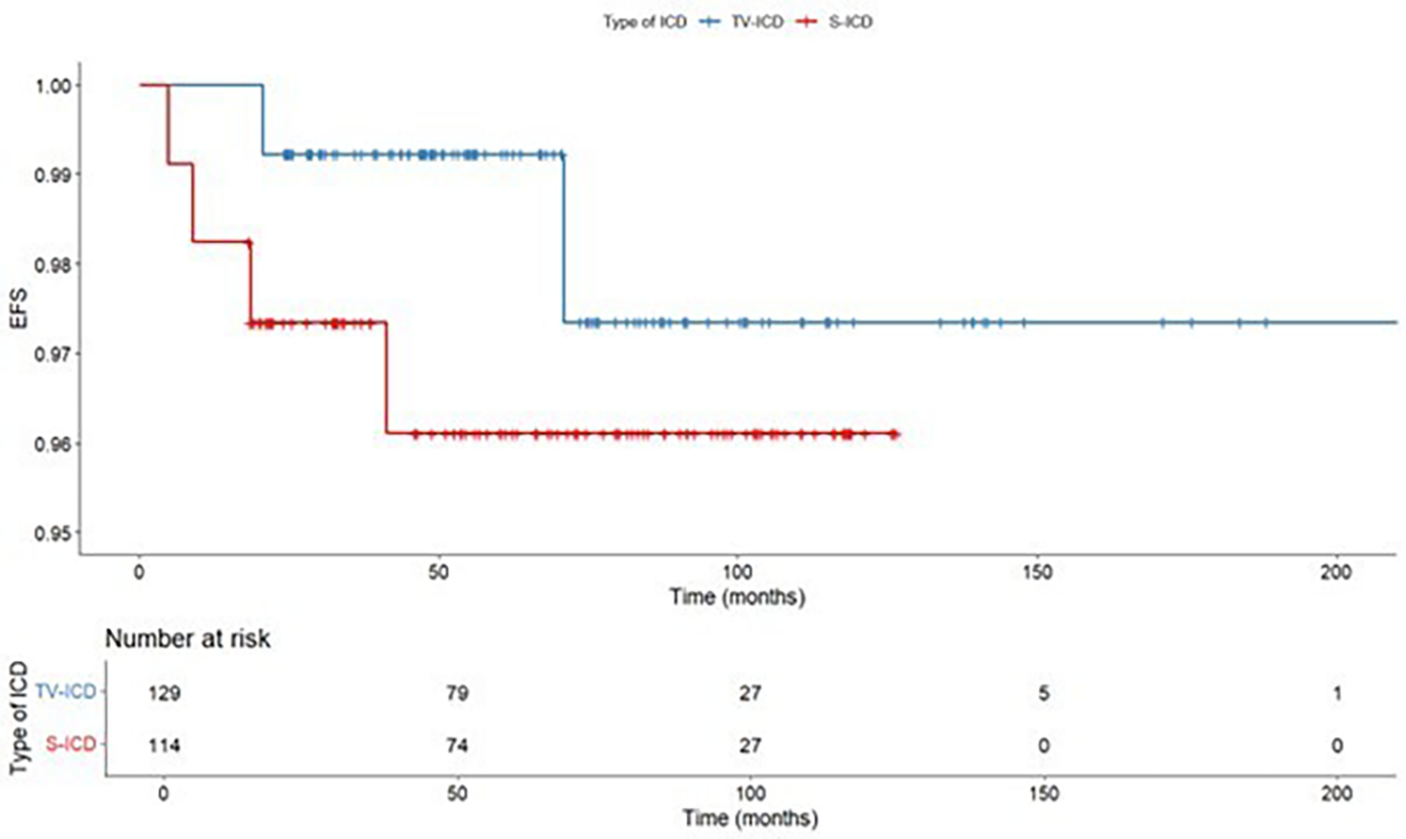
Kaplan maier curve for ICD related infections stratified according to ICD type.

ICD-related infections occurred in 8 patients (3.3%), of them, 1 (0.9%) in S-ICD group and 7 (5.4%) in the TV-ICD group (*p* = 0.048) (Table 3). The Kaplan–Meier analysis did not show a significantly different risk of ICD-related infections between the two subgroups (log-rank *p* = 0.055) (Figure 4).

All infected TV-ICD patients underwent lead extraction and subsequent S-ICD implantation; in two cases, a combined pacemaker leadless implantation was performed. The infected S-ICD patient was not re-implanted due to the absence of indication at clinical re-assessment after extraction.

At Cox multivariate analysis, a history of stroke/TIA [hazard ratio (HR) 7.77; 95% CI: 1.39–43.42; *p* = 0.020] and previous valve replacement (HR: 5.84; 95% CI: 1.25–27.28; *p* = 0.025) were independently associated with ICD infections, whereas S-ICD implantation was not (Supplementary Table S3).

## Discussion

The main findings of our study are as follows: (1) Among patients with ICM, no significant differences were observed in inappropriate ICD therapies or ICD-related complications between S-ICD and TV-ICD. However, TV-ICD was associated with a numerically higher, though not statistically significant, rate of ICD-related infections during follow-up compared to S-ICD. (2) Female gender and primary prevention were the only clinical factors independently associated with S-ICD implantation in ICM patients.

S-ICD is an established therapy for SCD prevention and an alternative to TV-ICD system in selected patients (1, 2). S-ICD showed a non-inferiority vs. TV-ICD for device-related complications or inappropriate shocks in patients with an indication for defibrillator therapy and not in need of pacing or CRT (3–10). Recently, two real-world registries showed S-ICD may be a valuable alternative to TV-ICD in patients with cardiomyopathies (16) and in those with heart failure (17); however, the potential risk of IAS, mainly due to non-cardiac oversensing, was not negligible.

Among different studies comparing the efficacy and safety of S-ICD vs. TV-ICD, the percentage of patients with ICM ranged from 27% to 67% (5, 9). No sub-analysis of randomized clinical trials including this subset of patients are currently available. In the EFFORTLESS registry (13), which included 28.1% of S-ICD patients with ICM, the ischemic etiology was an independent predictor of treated episodes for monomorphic ventricular tachycardia at five years. In a single-center retrospective study by Willy et al. (18) which included 45 patients with ischemic heart disease and S-ICD for primary or secondary prevention, no change to transvenous ICDs for anti-tachycardia pacing delivery was necessary, moreover, no surgical revision was required, and no system-related infections were reported during a mean follow-up of 2.5 ± 8.3 months. In an international observational study on 1,698 patients, of whom 31.7% had ischemic cardiomyopathy, no differences emerged between ischemic and non-ischemic patients regarding ICD appropriate shocks and device-related complications. However, ischemic patients showed a reduced risk of inappropriate ICD therapies (19).

Our data confirms in a large population the previous findings about the safety of S-ICD in patients with ICM. Among our study population, the cumulative incidence of inappropriate therapies was lower than previously reported (19, 20), mainly due to our strategy to optimize the TV-ICD programming at each follow-up visit or based on remote monitoring reporting. Moreover, the generation S-ICD systems implanted at our Institution have an additional high-pass filter to the sensing methodology, called SmartPass (SP), designed to reduce the inappropriate therapies (21, 22).

Among S-ICD patients, the air entrapment has been recently described as undetected cause of inappropriate therapies in the early post-procedural period (23–25). Regarding the complications, we observed a numerically reduction of overall ICD-related complications in the S-ICD group, mainly driven by less frequent lead-related complications. The low annual rate of ICD infections at our Institution confirms the reduced number of infections in high implantation volume centers (26); as we expected, the TV-ICD group showed a numerically higher incidence compared to the S-ICD group. These data may be explained by the multiprongest strategies we apply to reduce the cardiovascular implantable electronic device (CIED) infection, including the proper patients' selection, the basic preparation of the operating theater; the efforts to reduce hematoma formation; the use of an antibiotic-impregnated mesh envelope or antimicrobial solution during implantation in high-risk individuals. This evidence is of pivotal importance since systemic infections represent an important predictor of death for all causes, regardless of the result of the extraction procedure (27).

Among ICM patients, our data suggest a tendency to consider S-ICD the preferred choice for female patients and those in primary prevention. No data are available about the gender impact on the choice of ICD type. However, previous studies support for gender disparities in quality of life among ICD patients, with female patients reporting poorer mental health and more anxiety (28–31).

This preference for S-ICD in female patients may be explained by efforts to reduce the aesthetical impact of the TV-ICD wound in the anterior subclavian position, preferring instead for the more posterior and cranial placement of the S-ICD, where it minimally interferes with the position of the bra (32). Female S-ICD recipients experienced less likely appropriate ICD therapy, with similar risk of device-related complications compared to males; moreover, they were more likely to be at a low-risk of ventricular arrhythmias conversion failure (33).

Among our study population, the history of stroke/TIA and previous valve replacement were independently associated with ICD infections. According to the current guidelines, patients with prosthetic heart valves arec onsidered at high risk of infective endocardites (IE) and those receiving a CIED are considered at an intermediary IE risk (34). The combination of undergoing an prosthetic valve replacemente and having or getting a CIED may result in an even higher risk of IE, independently from the timing of the CIED implantation (35).

The evidence that previous stroke/TIA is a risk factor for CIED infection might be related to the type and magnitude of loss of function following the acute event. In a systematic review by Martino et al. the dysphagia occurs in 37%–78% of stroke patients and increases the risk for pneumonia 3-fold and 11-fold in patients with confirmed aspiration (36). In addition, a stroke may lead to an induced immunodepression, a systemic anti-inflammatory response that is related to susceptibility to infection (37, 38).

In clinical practice, the use of S-ICD in patients with ICM who do not require pacing or CRT remains challenging. This is primarily due to concerns about the potential for sustained VT that may require ATP or the risk of incident bradyarrhythmias that could necessitate pacing (39). However, it should be noted that only 15–20% of patients experienced a high rate of monomorphic VT during the first year after the implant with a subsequent risk is 1.8%/year; moreover, the proportion of both monomorphic VT and successful ATP was comparable between patients with ischemic and non-ischemic cardiomyopathy (40). Finally, no studies have still addressed whether the efficacy of ATP translates into hard outcomes such as mortality benefits, prevention of inappropriate shocks, and risks of pro-arrhythmias (41). Patients with ischemic cardiomyopathy had significantly less inappropriate therapy compared to patients with non-ischemic cardiomyopathy and appear to be appropriate patients for this type of device (39, 40). Moreover, patients with ischemic heart disease are particularly exposed to the risk of CIED-related complications due to their multiple comorbidities. This highlights the need for a patient-centered, tailored approach to device selection, rather than relying solely on the etiology of cardiomyopathy (ischemic vs. non-ischemic). Such an approach should consider not only the potential mechanisms of ventricular arrhythmias but also other patient-specific factors, including susceptibility to systemic infections, the concomitant use of other cardiac devices (42) and the risk of long-term device-related complications.

In the clinical contest of TV-ICD explanation, S-ICD has proven to offer a viable alternative for both infection and lead failure, since the S-ICD recipient mortality did not appear to be correlated with the presence of a prior infection, S-ICD therapy (appropriate or inappropriate), or S-ICD complications but rather to worsening of HF or other comorbidities (43, 44). Moreover, advancements in modular pacing-defibrillator systems offer promising solutions for patients requiring antitachycardia or bradycardia pacing. A recently developed system combines a leadless pacemaker with a subcutaneous ICD, enabling wireless communication to provide both pacing modalities. Early data have demonstrated freedom from major complications related to the leadless pacemaker and its communication with the S-ICD. Furthermore, at six months, the majority of patients achieved adequate pacing thresholds, with a pulse width of 0.4 ms and a pacing voltage of up to 2.0 V (30). This innovation underscores the potential for further improving outcomes in this complex patient population by combining the benefits of S-ICD with advanced pacing technologies (45).

### Study limitations

Our results should be interpreted considering the limitations related to the study's retrospective, observational, and single-center nature; however, it is the largest study evaluating the clinical performance of S-ICD vs. TV-ICD among patients with ischemic cardiomyopathy not in need of pacing or CRT. The findings of our study may be influenced by the high level of experience in ICD implantation and management at our center. The follow-up duration is relatively short, approximately 65 months; however, it remains the longest observational study including this subset of patients. Additionally, no data on pharmacological therapies or biomarkers were collected at the time of outcome events (46). An additional limitation is the small number of patients undergoing ventricular tachycardia ablation (4 in the TV-ICD group and 1 in the S-ICD group), which precluded meaningful analysis of its impact. This contrasts with findings from Schiavone et al., who reported improved long-term outcomes, including reduced arrhythmic events and cardiovascular mortality, in S-ICD carriers undergoing ablation (47). Furthermore, the associations between female sex and primary prevention as independent predictors of S-ICD implantation warrant further investigation in a multicenter study, ideally including comparisons with patients with non-ischemic cardiomyopathy, to better assess their broader applicability and clinical significance.

## Conclusions

In our clinical practice, the decision to implant an S-ICD in ICM patients was mainly driven by female sex and primary SCD prevention. No significant difference in inappropriate ICD therapies or ICD-related complications has been observed between TV-ICD and S-ICD; even if these latter showed a numerically lower risk of ICD-related infections. Our findings suggest that S-ICD may be a viable alternative to TV-ICD in ICM patients; however further prospective randomized studies are needed to confirm these results and explore their broader applicability.

## Data Availability

The data that support the findings of this study are available on reasonable request from the corresponding author.
