# Supplementary material for: Clinical performance of subcutaneous vs. transvenous implantable defibrillator in patients with ischemic cardiomyopathy: data from Monaldi Rhythm Registry
Source: Front Cardiovasc Med. 2025 Feb 19;12:1539125. doi: 10.3389/fcvm.2025.1539125 (PMC11880277; doi:10.3389/fcvm.2025.1539125)
Supplement: Supplementary file 1 [file Table1.docx]

**Supplementary Table 1.** Univariable and multivariable Cox regression model for inappropriate ICD therapy

|  | **Univariable Analysis** | | | | **Multivariable Analysis** | | | |
| --- | --- | --- | --- | --- | --- | --- | --- | --- |
| **Parameter** | **HR** | **95% CI** | | **P** | **HR** | **95% CI** | | **p** |
| **Age** | 0.94 | 0.88 | 0.99 | **0.032** | 0.97 | 0.88 | 1.08 | 0.607 |
| **Sex**  *M*  *F* | 1  1.02 | 0.21 | 5.08 | 0.977 |  |  |  |  |
| **S-ICD** | 2.31 | 0.42 | 12.64 | 0.333 |  |  |  |  |
| **Primary prevention** | 1.41 | 0.16 | 12.07 | 0.754 |  |  |  |  |
| **Secondary Prevention** | 0.71 | 0.08 | 6.06 | 0.754 |  |  |  |  |
| **NYHA class>2** | 0.08 | 0.02 | 0.34 | **<0.001** | 0.37 | 0.06 | 2.12 | 0.262 |
| **LVEF** | 1.13 | 1.05 | 1.21 | **<0.001** | 1.02 | 0.91 | 1.14 | 0.741 |
| **Hypertension** | 0.21 | 0.04 | 1.13 | 0.070 | 0.34 | 0.04 | 2.70 | 0.306 |
| **Diabetes** | 0.00 | 0.00 | ∞ | 0.957 |  |  |  |  |
| **COPD** | 0.57 | 0.07 | 4.92 | 0.613 |  |  |  |  |
| **CAD history** | 0.19 | 0.04 | 0.94 | **0.041** | 0.45 | 0.05 | 3.95 | 0.494 |
| **Previous CABG** | 0.83 | 0.10 | 7.10 | 0.863 |  |  |  |  |
| **Previous PTCA** | 0.15 | 0.02 | 1.25 | 0.075 | 0.36 | 0.03 | 3.95 | 0.691 |
| **History of Stroke/TIA** | 0.00 | 0.00 | ∞ | 0.965 |  |  |  |  |
| **PAD** | 0.00 | 0.00 | ∞ | 0.961 |  |  |  |  |
| **CKD** | 0.67 | 0.08 | 5.76 | 0.718 |  |  |  |  |
| **AF history** | 5.76 | 1.05 | 31.46 | **0.043** | 4.62 | 0.76 | 28.22 | 0.097 |
| **Previous valve replacement** | 0.00 | 0.00 | ∞ | 0.957 |  |  |  |  |

ICD implantable cardioverter defibrillator; S-ICD subcutaneous implantable cardioverter defibrillator; LVEF left ventricular ejection fraction; COPD chronic obstructive pulmonary disease; CAD coronary artery disease; CABG coronary artery bypass graft; PTCA percutaneous transluminal coronary angioplasty; TIA transient ischemic attack; PAD peripheral artery disease; CKD chronic kidney disease; AF atrial fibrillation

**Supplementary Table 2.** Univariable and multivariable Cox regression model for ICD related complications

|  | **Univariable Analysis** | | | | **Multivariable Analysis** | | | |
| --- | --- | --- | --- | --- | --- | --- | --- | --- |
| **Parameter** | **HR** | **95% CI** | | **P** | **HR** | **95% CI** | | **p** |
| **Age** | 0.98 | 0.92 | 1.03 | 0.393 |  |  |  |  |
| **Sex**  *M*  *F* | 1  0.50 | 0.12 | 1.99 | 0.499 |  |  |  |  |
| **S-ICD** | 0.32 | 0.07 | 1.55 | 0.158 |  |  |  |  |
| **Primary prevention** | 1.00 | 0.21 | 4.82 | 0.999 |  |  |  |  |
| **Secondary prevention** | 1.00 | 0.21 | 4.82 | 0.999 |  |  |  |  |
| **NYHA class>2** | 2.31 | 0.90 | 5.98 | 0.083 | 2.28 | 0.87 | 6.01 | 0.094 |
| **LVEF** | 0.91 | 0.82 | 1.01 | 0.068 | 2.95 | 0.37 | 23.60 | 0.308 |
| **Hypertension** | 3.16 | 0.40 | 25.26 | 0.278 |  |  |  |  |
| **Diabetes** | 0.61 | 0.13 | 2.96 | 0.543 |  |  |  |  |
| **COPD** | 1.47 | 0.37 | 5.87 | 0.587 |  |  |  |  |
| **CAD history** | 0.00 | 0.00 | ∞ | 0.962 |  |  |  |  |
| **Previous CABG** | 0.58 | 0.07 | 4.63 | 0.580 |  |  |  |  |
| **Previous PTCA** | 2.62 | 0.54 | 12.59 | 0.230 |  |  |  |  |
| **History of Stroke/TIA** | 0.00 | 0.00 | ∞ | 0.969 |  |  |  |  |
| **PAD** | 2.20 | 0.46 | 10.58 | 0.326 |  |  |  |  |
| **CKD** | 1.68 | 0.42 | 6.73 | 0.461 |  |  |  |  |
| **AF history** | 0.34 | 0.04 | 2.71 | 0.307 |  |  |  |  |
| **Previous valve replacement** | 0.00 | 0.00 | ∞ | 0.964 |  |  |  |  |

ICD implantable cardioverter defibrillator; S-ICD subcutaneous implantable cardioverter defibrillator; LVEF left ventricular ejection fraction; COPD chronic obstructive pulmonary disease; CAD coronary artery disease; CABG coronary artery bypass graft; PTCA percutaneous transluminal coronary angioplasty; TIA transient ischemic attack; PAD peripheral artery disease; CKD chronic kidney disease; AF atrial fibrillation

**Supplementary Table 3.** Univariable and multivariable Cox regression model for ICD-related infections

|  | **Univariable Analysis** | | | | **Multivariable Analysis** | | | |
| --- | --- | --- | --- | --- | --- | --- | --- | --- |
| **Parameter** | **HR** | **95% CI** | | **P** | **HR** | **95% CI** | | **p** |
| **Age** | 0.96 | 0.91 | 1.02 | 0.191 |  |  |  |  |
| **Sex**  *M*  *F* | 0.34 | 0.07 | 1.68 | 0.184 |  |  |  |  |
| **S-ICD** | 0.16 | 0.02 | 1.34 | 0.092 | 0.16 | 0.02 | 1.36 | 0.093 |
| **Primary prevention** | 0.48 | 0.11 | 2.00 | 0.311 |  |  |  |  |
| **Secondary prevention** | 2.10 | 0.50 | 8.79 | 0.311 |  |  |  |  |
| **NYHA class>2** | 1.31 | 0.47 | 3.65 | 0.610 |  |  |  |  |
| **LVEF** | 0.96 | 0.88 | 1.06 | 0.426 |  |  |  |  |
| **Hypertension** | 1.25 | 0.25 | 6.22 | 0.783 |  |  |  |  |
| **Diabetes** | 1.33 | 0.32 | 5.55 | 0.700 |  |  |  |  |
| **COPD** | 0.40 | 0.05 | 3.24 | 0.389 |  |  |  |  |
| **CAD history** | 1.38 | 0.17 | 11.23 | 0.762 |  |  |  |  |
| **Previous CABG** | 1.50 | 0.30 | 7.42 | 0.622 |  |  |  |  |
| **Previous PTCA** | 0.24 | 0.05 | 1.20 | 0.082 | 0.36 | 0.07 | 1.96 | 0.239 |
| **History of Stroke/TIA** | 8.46 | 1.70 | 42.05 | **0.009** | 7.77 | 1.39 | 43.42 | **0.020** |
| **PAD** | 0.00 | 0.00 | ∞ | 0.955 |  |  |  |  |
| **CKD** | 3.44 | 0.86 | 13.78 | 0.081 | 3.34 | 0.75 | 14.82 | 0.112 |
| **AF history** | 0.94 | 0.19 | 4.64 | 0.936 |  |  |  |  |
| **Previous valve replacement** | 10.08 | 2.41 | 42.20 | **0.002** | 5.84 | 1.25 | 27.28 | **0.025** |

ICD implantable cardioverter defibrillator; S-ICD subcutaneous implantable cardioverter defibrillator; LVEF left ventricular ejection fraction; COPD chronic obstructive pulmonary disease; CAD coronary artery disease; CABG coronary artery bypass graft; PTCA percutaneous transluminal coronary angioplasty; TIA transient ischemic attack; PAD peripheral artery disease; CKD chronic kidney disease; AF atrial fibrillation
